# Issues and Prospects of microRNA-Based Biomarkers in Blood and Other Body Fluids

**DOI:** 10.3390/molecules19056080

**Published:** 2014-05-14

**Authors:** John R. Chevillet, Inyoul Lee, Hilary A. Briggs, Yuqing He, Kai Wang

**Affiliations:** 1 Institute for Systems Biology, Seattle, WA 98109, USA; 2 Institute of Medical Systems Biology, Guangdong Medical College, Dongguan, Guangdong 523808, China; 3 Laboratory of the Biology of Addictive Diseases, Rockefeller University, New York, NY 10065, USA

**Keywords:** diagnostic, exosomes, microvesicles, quantitative methods

## Abstract

Cell-free circulating microRNAs (miRNAs) in the blood are good diagnostic biomarker candidates for various physiopathological conditions, including cancer, neurodegeneration, diabetes and other diseases. Since their discovery in 2008 as blood biomarkers, the field has expanded rapidly with a number of important findings. Despite the initial optimistic views of their potential for clinical application, there are currently no circulating miRNA-based diagnostics in use. In this article, we review the status of circulating miRNAs, examine different analytical approaches, and address some of the challenges and opportunities.

## 1. Introduction

MicroRNAs (miRNAs) were originally identified as small, non-coding RNA mediators of temporal pattern formation in *C. elegans* [1,2,3,4,5], which regulated messenger RNAs (mRNAs) by base pairing with their 3'-untranslated regions (3'-UTR). Later research showed that miRNAs (and this type of RNA mediated regulatory processes) are conserved in metazoan species [6,7,8,9] and have also been found in viruses [10]. MiRNAs are currently differentiated from other small RNAs by: (1) a hairpin fold-back precursor structure in their primary transcript, as predicted by base composition (2) and an approximately 18–22 nucleotide long mature sequence [11]. At present, 1,872 precursor sequences and 2,578 mature human sequences have been catalogued by the online repository miRBase (v20) [12]. 

Primary miRNA transcripts (pri-miRNAs) are transcribed in the nucleus and processed by an RNase III enzyme, Drosha [13]. The resulting smaller hairpin pre-miRNA is transported to the cytoplasm via a RanGTP-dependent double stranded RNA binding protein, exportin 5. The pre-miRNA is then further processed by another RNase III, Dicer [14], into an approximately 22 nucleotide long duplex RNA with 3’ overhangs. The duplex is subsequently unwound by RNA helicase and usually one strand (the guide strand) is preferentially associated with the effector-protein Argonaute [15,16], as part of the RNA Induced Silencing Complex (RISC). The other strand (the passenger strand) is degraded. The process for strand selection is yet to be fully understood. The miRNA-RISC complex can then interact with mRNA targets to inhibit translation or induce degradation of the mRNA, typically through binding at the 3'-untranslated region (3'-UTR) with partial sequence complementarity. The RNase III cleavages are often imprecise, resulting in end region sequence variations (especially the 3' end) of mature miRNAs. In addition to this, miRNAs can be post-transcriptionally modified (e.g., via non-templated additions), resulting in sequence variants (isomiRs) [17,18,19,20], which may have functionally distinct properties [21,22,23], expanding the miRNA repertoire. 

Through interacting with mRNA targets, miRNAs predominantly act to modulate the transcriptome of cells [24] and have been implicated in various physiopathological processes, including development, homeostasis and cellular pathology [25,26]. Based on various prediction algorithms, the majority of mRNA transcripts are potential targets for miRNAs [27]. MicroRNA-target binding is usually mediated through the initial interaction of the seed region (nucleotides 2-8 of the 5' end of the miRNA) [13], but a significant fraction (approximately 60% based on one report [28]) of seed interactions are non-canonical (*i.e.*, contain bulging or mismatches) and are generally associated with additional, non-seed base pairing. These flexible constraints on specificity permit a single miRNA to target multiple (possibly hundreds) of mRNAs and elicit effects on multiple biological processes. A single mRNA can also contain binding sites for multiple different miRNA sequences. This makes miRNA-mediated transcriptome/proteome regulation a complicated process and difficult to decipher.

The expression of certain miRNAs is restricted to specific cell types [29] or developmental stages; therefore, they are frequently dysregulated in pathologies including cancer [30,31,32,33], cardiovascular diseases [34,35,36,37,38,39,40,41] and neurological conditions [42,43,44,45,46]. In the case of cancer, analyzing the miRNA spectrum of tumors yields qualitative diagnostic information and allows possible identification of the tissue of origin and in some cases, provides molecular stratification of tumor subtypes [47,48,49]. 

## 2. Early Studies of Circulating miRNA

The presence of cell-free nucleic acids in blood circulation has been established for almost 60 years [50,51,52,53,54]. Tumor derived DNA and RNA are commonly observed in cancer patient plasma samples [55,56,57,58]. RNA was historically thought to be unsuitable as a practical blood-based biomarker, due to the high level of nuclease activity in human plasma [59], but this skeptical position was reconsidered after the observation that miRNAs are stable in fixed tissues [60] and that the concentration of miR-21 in serum could distinguish patients with B-cell lymphoma from control individuals [61]. Subsequent work detected specific circulating miRNA signatures in healthy individuals and patients with prostate, breast and colorectal cancers [62,63,64,65,66,67], in addition to other disorders. Detailed examination [62] revealed that the detected species were *bona fide* cell-free miRNAs and explicitly demonstrated their stability outside of the cells (tolerating prolonged incubation at room temperature in addition to multiple cycles of freezing and thawing of the plasma samples). The release of miRNA from tumors was further demonstrated by detecting human miRNA sequences in plasma drawn from mice harboring human xenografts. The field expanded rapidly, describing numerous circulating miRNA biomarkers for cancer (reviewed elsewhere in this issue [68], and other conditions including neurodegeneration, cardiovascular disease and metabolic disease (Table 1). Although it has been demonstrated that miRNAs can be released into circulation from cells specific to the disease (e.g., miR-122 from liver toxicity studies [69,70]) the cellular origin of many miRNA in biofluids is unclear. For example, blood levels of miRNAs highly expressed in heart tissues, such as miR-208a and miR-499, were included as a part of signatures to differentiate patients with cardiovascular disease in several studies [71,72,73,74]. This suggests that increased levels of heart-specific blood miRNAs may derive from the heart and directly reflect the heart condition of patients. However whether all reported miRNAs are actually directly associated with the disease or are the product of nonspecific, systemic or secondary responses remains to be further evaluated. 

**Table 1:** **Circulating miRNA biomarkers for neurodegeneration, cardiovascular and metabolic diseases.** 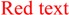: increased abundance relative to controls, 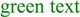: reduced abundance relative to controls. (AD) Alzheimer Disease, (CAD) Coronary Artery Disease, (GDM) Gestational Diabetes Mellitus, (HD) Huntington Disease, (IFG) impaired fasting glucose, (IGT) impaired glucose tolerance, (MCI) Mild Cognitive Impairment, (NEC) Normal Elderly Controls, (NGS) Next Generation Sequencing, (PBMC) Peripheral Blood Mononuclear Cells, (PD) Parkinson Disease, (T1D) Type 1 Diabetes, (T2D) Type 2 Diabetes, (RT-qPCR) Reverse Transcription Quantitative PCR.

Disease		miRNA	Detection Method	Specimen	Population	Ref.
**Neurodegenerative Disease**	Alzheimer Disease	miR-34a, -181b, -200a, let-7f	Microarray, RT-qPCR	PBMC	16 AD patients and 16 NEC matched for ethnicity, age, gender and education	[75]
		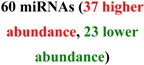	RT-qPCR	CSF	10 AD, 10 controls	[76]
		miRNA-9, -125b, -146a, -155	Microarray, Northern dot blot	CSF	6 AD, 6 controls	[77]
		miR-137, -181c, -9, -29a, -29b	RT-qPCR	Serum	7 AD, 7 controls	[78]
		miR-29b	RT-qPCR	PBMC	393 AD, 412 controls	[79]
		miR-15a	RT-qPCR	Plasma, CSF	11 AD, 9 MCI, 20 NC; 20 AD, 17 NC	[80]
		let-7d-5p, let-7g-5p, miR-15b-5p, -142-3p, -191-5p, -301a-3p, -545-3p	nCounter, RT-qPCR	Plasma	Screening: 11 AD, 20 NC; validation 20 AD, 17 NC	[81]
		miR-34c	RT-qPCR	Plasma	110 AD, 123 NEC	[82]
		miR-146a	RT-qPCR	CSF	10 AD, 10 early AD, 11 controls	[83]
	Parkinson Disease	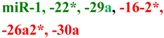	RT-qPCR	Whole blood	15 PD (8 untreated PD), 8 controls	[84]
		18 miRNAs	Microarray	PBMC	19 PD, 13 controls	[85]
		miR-222, -626, -505	Microarray, RT-qPCR	Plasma	32 PD, 34 controls; 42 PD, 30 controls; 30 PD, 8 controls	[86]
		miR-331-5p	RT-qPCR	Plasma	31 PD, 25 controls	[87]
		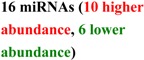	NGS	Blood leukocyte	7 PD, 6 controls	[88]
	Huntington Disease	miR-34b	Microarray, RT-qPCR	Plasma	27 HD, 12 controls	[89]
**Cardiovascular Disease**	Coronary Artery Disease	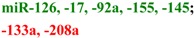	RT-qPCR	Plasma, serum	8 CAD, 8 controls; 36 CAD, 17 controls	[71]
		miR-135a and miR-147	RT-qPCR	PBMC	50 CAD, 20 controls	[90]
		miR-146a and miR-146b	RT-qPCR	PBMC	41 CAD, 15 controls	[91]
		miR-19a, -584, -155, -222, -145, -29a, -378, -342, -181d, -30e-5p, -150)	Microarray RT-qPCR	Whole blood	5 CAD, 5 controls for initial; 10 CAD, 15 controls for validation	[92]
		miR-214	RT-qPCR	Plasma	40 CAD, 15 controls	[93]
	Acute Myocardial Infarction	miR-1, -133a, -499, -208a	Microarray, RT-qPCR	Plasma	33 AMI, 30 controls	[72]
		miR-1	RT-qPCR	Plasma	93 AMI, 66 controls	[94]
		miR-1	RT-qPCR	Serum	31 AMI, 20 controls	[95]
		miR-499	Microarray, RT-qPCR	Plasma	14 AMI, 15 heart failure patients, 10 controls	[73]
		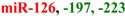	Microarray, RT-qPCR	Plasma	820 Bruneck cohort [96]	[97]
		miR-1915, -181	RT-qPCR	Whole blood	60 AMI, 21 controls, 5 time points (0–24 h)	[98]
		miR-133a	RT-qPCR	Plasma	13 AMI patients, 176 angina pectoris patients, 127 controls	[99]
		miR-1, -134, -186, -208, -223 and -499	NGS, RT-qPCR	Serum	117 AMI patients, 182 AP patients, 100 controls	[74]
	Congestive Heart Failure	miR-210	RT-qPCR	PBMC	13 patients, 6 controls	[100]
		miR-126	RT-qPCR	Plasma	33 patients, 17 controls	[101]
	Aortic Aneurysm	miR-29b, -124, -155, -223	RT-qPCR	Plasma	23 patients, 12 healthy controls, 17 coronary artery disease patients	[102]
	Stroke	miR-125b-2*, -27a, -422a, -488, -627	Microarray, RT-qPCR	Plasma	169 stroke patients, 94 metabolic syndrome patients, 24 healthy controls	[103]
		miR-145	RT-qPCR	Whole Blood	32 ischemic stroke patients, 14 healthy controls	[104]
	Atherosclerosis	miR-130a, -27b, -210	RT-qPCR	Serum	104 patients, 105 controls	[105]
**Metabolic Disease**	Type 1 Diabetes	miR-152, -30a-5p, -181a, -24, -148a, -210, -27a, -29a, -26a, -27b, -25, -200a	NGS, RT-qPCR	Serum	pooled from 2 T1D groups (275, 129) and one control group (*n* = 151)	[106]
	Type 2 Diabetes	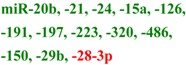	Microarray, RT-qPCR	Plasma	80 patients, 80 controls	[107]
		miR-9, -29a, -30d, -34a, -124a, -146a and -375	RT-qPCR	Serum	18 T2D, 19 pre-diabetes (IGT and/or IFG), 19 controls	[108]
		miR-146a	RT-qPCR	Plasma	90 patients, 90 controls	[109]
		miR-29a	RT-qPCR	Urine	83 patients (42 with albuminuria, 41 with normoalbuminuria)	[110]
		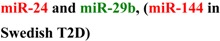	RT-qPCR	Plasma	33 patients (14 Swedes, 19 Iraqis), 119 controls	[111]
	Gestational Diabetes Mellitus	miR-132, -29a and miR-222	RT-qPCR	Serum	24 GDM, 24 controls	[112]

## 3. Physical State and Biological Function

The stability of cell-free circulating miRNA in the extracellular environment (e.g., plasma) had been established, but the mechanistic basis of this resistance to degradation remained unclear. Synthetic miRNA oligonucleotides and miRNA purified from plasma samples were rapidly degraded when combined with non-denatured plasma [62], indicating that miRNAs were not stabilized by intrinsic factors such as secondary structure or chemical modification. This observation led to the hypothesis in the field that cell-free circulating miRNAs were packaged in a way to protect them from RNase degradation, possibly in lipid vesicles or as part of a protein complex.

### 3.1. Exosomes and Other Vesicles

Among extracellular lipid vesicles, exosomes have received considerable attention [113,114,115,116]. Exosomes are 50–100 nm diameter membrane vesicles, secreted by diverse cell types *in vivo* and *in vitro*. These vesicles are present in many bodily fluids (e.g., blood, saliva, breast milk, seminal fluid, ascites, urine, *etc.*) [115,117], and have been shown to contain characteristic protein, mRNA and miRNA molecules derived from their cell of origin. In addition to exosomes, cells also produce larger lipid vesicles, microvesicles (1–10 μm in diameter). Like exosomes, microvesicles also contain protein, miRNA and mRNA. Recently, prostate cancer cells have been observed to produce microvesicles, which have been termed large oncosomes (LOs) [118]. LOs contain miRNAs associated with cell migration and invasion. Apoptotic bodies derived from normal cell turn over or from specific pathologies (e.g., cancer) may also contribute to RNA in circulation. 

Circulating miRNAs may also be derived from platelets, which are abundant in typical plasma preparations, contain a large quantity of miRNA [119,120] and have been reported to contain tumor-derived mRNA when isolated from the plasma of cancer patients [121]. The authors of this study suggest that platelets acquire tumor-derived mRNA through exosome-mediated transfer, although it is possible that platelet preparations may be contaminated with circulating tumor cell debris [122].

Several studies have observed the uptake of exosomes and other miRNA-containing vesicles into cells *in vitro* [116,118,123,124,125], leading to the hypothesis of miRNA-based intercellular communication mediated by exosomes (like a “message in a bottle” [126]) but it remains unclear if this is a general phenomenon.

### 3.2. Protein-miRNA Complexes

Multiple studies have also reported cell-free circulating miRNAs in non-lipid vesicle forms [127,128]. Using differential centrifugation, size-exclusion chromatography, filtration and immunoprecipitation approaches, investigators observed that a large fraction of miRNAs in platelet-poor plasma/serum from healthy human donors and conditioned cell medium were not associated with lipid vesicles and were sensitive to protease digestion. This observation suggested that miRNA was protected from plasma RNase activity by association with protein. A substantial amount of this soluble miRNA was associated with the endogenous effector protein Argonaute-2 (Ago2). Independent studies have also observed miRNA associated with other proteins such as high-density lipoprotein [129] and nucleophosmin [130]. 

### 3.3. Exogenous miRNAs in Circulation

Recent reports describing exogenous miRNA and other RNA species in the blood of healthy individuals [131,132] have raised some interesting questions about the genesis, function, and complexity of cell-free RNA in circulation. To accurately determine the origin of these RNAs derived from exogenous species is a challenge, due to the lack of genomic sequence information for many different species. Nevertheless, the preliminary results indicate these RNA molecules are probably derived from numerous bacteria, fungi and food, which suggest that gut is the primary source of these sequences. Functional delivery of small RNAs through ingestion has been very well established in nematodes [133]. However, tissue barrier systems in mammals are much more elaborate and it is unclear how RNA might penetrate them. Limited experimental data also showed that some of these exogenous RNAs are packaged in lipid vesicles and can affect the cellular transcriptome upon entry into the cells. How these RNAs permeated the gut lining, how they are packaged, how they are selected and how did cells recognize and take up these exogenous RNA species are just some of the interesting issues are waiting to be answered. The finding of exogenous miRNAs in normal human blood also raises some interesting concerns. Since some miRNA sequences are extremely conserved throughout evolution, the levels of those miRNAs in circulation may not be completely contributed from human cells. In addition, pathogenic or adverse effects induced by exogenous RNAs are a possibility. 

## 4. Status of Circulating miRNA Biomarkers

Since the establishment of circulating miRNAs as biomarkers for cancer in 2008 [61,62], there have been over 500 reports describing their biology and diagnostic utility for various conditions. Relative to other types of diagnostic biomolecules in circulation (e.g., proteins), miRNA has many advantages including tissue or cell type/stage specific expression, mature nucleic acid detection technologies, lower cost and shorter time required to develop assays (compared to the development of proteomic tools such as antibodies) and amplifiable signal. Despite these advantages, the rapid expansion of the literature, and significant commercial interest, circulating miRNA biomarkers face an unclear path toward clinical application. For example, an initial study of a circulating miRNA panel on 120 patients provided 91 percent sensitivity and 72 percent specificity to diagnose colon cancer. However, later studies yielded inconsistent results [134]. The inconsistencies were attributed to variation in specimen collection and handling, which are well known to alter miRNA spectrum [120]. Additional factors might also have been involved.

## 5. Considerations for Circulating MiRNA Studies

### 5.1. Prerequisites

Ideal prerequisites for biomarker discovery/development processes should include: (1) the need for such biomarker, (2) the intended use (e.g., type of tumor *vs.* tumor progression or response to treatment), (3) well characterized patient and matched control specimens in acceptable quantity, (4) samples have been banked or can be prospectively collected, processed and stored in a consistent manner and (5) a specific, reliable and easy to implement detection method. This process has been done for other clinical tests such as pathogen screening and protein-based biomarker measurement, but there is no commonly accepted standard for miRNA. In part this is due to the lack of understanding of both intrinsic and extrinsic factors that may affect the miRNA spectrum. Therefore, fundamental and comprehensive studies are urgently needed to address these issues. This need is well recognized in the community. For example, the National Institutes of Health recently established the Extracellular RNA Communication (ERC) program [135] to explore various properties and applications for extracellular miRNA in circulation.

### 5.2. Sample type, Collection and Processing

Donor-related factors such as the feeding state (e.g., fasting *vs.* non-fasting, food lipid content, *etc.*), time of blood draw, gender, and female hormone cycle may all affect the spectrum of circulating miRNA. However, the impact of these factors is largely unknown at this point. The type of blood product analyzed (serum *vs.* plasma) has also been shown to affect the spectrum of circulating miRNA [136]. Several known issues associated with the practice of blood collection and serum/plasma preparation are listed below:

- The use of small diameter needles (23 gauge or above) should be avoided due to shearing induced hemolysis of red blood cells (RBCs) [137], which contain abundant miRNAs and can alter profiling results [119,138,139].
- Lot-to-lot variation in the production of blood collection tubes may influence results [140]; if possible, purchase draw tubes from the same lot. Discard expired tubes, as these may have lost vacuum and cause variation on the final concentration of anticoagulant in the blood.
- Skin contains abundant epithelial miRNAs; thus a precursor blood draw or a blood tube that is drawn and discarded [141] using the same needle and tubing can prevent skin cells from contaminating a blood sample (especially for studies of epithelial cancers).
- Anticoagulant choice is also critical; this should be based on the requirements for analysis and uniform across the study (using only one type of anticoagulant tube with the part number and vendor specified in the protocol). Heparin is a well-established inhibitor of PCR and should be avoided [142]; EDTA tubes have been widely used instead. In the event heparin-containing samples are required, heparinase treatment prior to analysis has been shown to increase miRNA detection [143] but is likely to introduce additional variability.
- Anticoagulants and blood samples should be gently mixed in the tube, as shaking can cause hemolysis [144]. Serum coagulation conditions (time, temperature) and use of serum-separator polymers should be standardized between cases and controls.
- Blood should be expediently processed and the time allowed between draw and processing should be stated in the protocol. Specimens exceeding this time limitation should be flagged and noted in the data analysis.
- Insulated containers with uniform temperature should be used in packing specimens for transit. If environmental conditions include possible extreme heat or cold exposure, devices that can indicate if the specimen has exceeded a threshold temperature should be used [140].
- Standardized centrifugation conditions used to prepare cell-free blood fractions (time, temperature, *g*-force, rotor type, acceleration/deceleration conditions) are also important as residual platelets, cell debris, *etc.* can alter miRNA abundance [120]. Blood fractions require expedient separation from cell pellets to prevent contamination with cellular debris and contents [138,145].
- As blood cells contain higher concentrations of miRNAs than plasma, care should be taken in aspirating plasma and serum to prevent cellular carryover [146].
- Centrifugation to remove debris or precipitates from body fluid samples prior to RNA extraction needs to be standardized, as it will alter miRNA profiles.
- To avoid unnecessary freeze/thaw cycles, if possible samples should be stored as single use aliquots, with volumes corresponding to those intended for analysis.
- Freezing and storage conditions should be standardized (snap or slow freezing). In addition, case/control specimens should be matched as closely as possible for storage time in the freezer.

### 5.3. RNA Isolation

The method used to purify RNA impacts yield and the spectrum of RNA isolated [142,147]. Methods based on phenol-chloroform extraction and silica-membrane based columns have been observed to enhance detection of circulating miRNAs relative to methods that omit either of these steps. Omission of chloroform extraction may be especially problematic for blood samples containing high lipid content (e.g., blood drawn after high-fat meals, *etc.*). miRNA-specific methods are important, as buffers for miRNA extraction often utilize higher ethanol concentrations than their mRNA counterparts. Whether or not to enrich small RNA fraction is yet to be determined, since there are reports indicating the alteration of small RNA profile after enrichment. Addition of carrier RNA (e.g., MS2 bacteriophage RNA) [148] has been observed to improve miRNA quantification, but more data is needed to demonstrate its reliability. In addition, carrier RNA may also affect downstream profiling methods (e.g., next-generation sequencing). The use of low-adhesion plasticware is critical, as standard plastics can adsorb miRNAs and skew quantification. 

MiRNA derived from exosomes and other vesicles has been a significant focus of study, however; the purification method used to isolate vesicles will impact the type of vesicles that are collected and therefore their miRNA content. Isolation of lipid vesicles is generally performed via differential centrifugation [149,150,151]. The centrifugation force, type of rotor (e.g., fixed angle *vs.* swinging bucket) and viscosity of the sample are important variables. Exoquick (EXOQ5A-1, System Biosciences, Mountain View, CA), a polymer solvent, facilitates precipitation of exosomes [152,153] but may also precipitate non-exosomal vesicles and protein-miRNA complexes [154]. 

### 5.4. Data Correction, Normalization, Standards and Avoiding Contamination

Controls for variation in sample quality, processing and analysis can be introduced throughout the workflow. Hemolysis can be estimated using colorimetry (less sensitive) of the blood product specimens or by analysis of miRNAs abundant in RBCs (e.g., miR-451) [119,155]. Normalization to correct for variability during sample purification is typically achieved through the use of synthetic oligoribonucleotides (with low homology to those expressed in the species of interest) spiked-in to the samples after thorough denaturation [146]. Additional spike-in standards (e.g., UniSp6 and UniSp3, Exiqon) can be introduced prior to cDNA synthesis and PCR to estimate efficiency during these steps and normalize results for comparison. 

Standard curves are typically prepared by serial dilution of synthetic oligoribonucleotide standards. To ensure reproducible quantification, these standards should be prepared as single use aliquots in low-adhesion plastics. The choice of diluent in which to prepare the standard curve is important, as the solution matrix alters the performance of the assay significantly [156] through effects on analyte recovery from liquid transfers, enzyme and primer efficiencies, *etc.* The most appropriate choice is a diluent that approximates the complexity of the samples (e.g., total RNA prepared from plasma) or the use of an abundant carrier RNA to dominate matrix effects (e.g., MS/2 bacteriophage RNA) [148]. It is possible that assays may cross-react with sequences present in the carrier RNA; thus, the tolerance of the assay for the carrier should be empirically determined. 

As the concentration of standards and analytes span several orders of magnitude, care must be taken to avoid cross contamination, especially with concentrated stocks of synthetic standards, target-rich biologic samples and post-PCR products. Contamination events have proven a serious distraction for blood-based molecular diagnostics [157,158,159]. Analytic layouts should include multiple no-template controls distributed over the analysis plate as sentinel wells, to detect possible aerosol contamination of wells during plate manipulation. Dedicated pre-PCR workspaces, equipment and protocols should be used [144,160,161].

### 5.5. Technologies for miRNA Profiling

A variety of methods have been developed for miRNA profiling [162,163], but due to their short sequence length, end region sequence variation (e.g., isomiRs) and high sequence conservation among family members, accurate measurement for miRNA is nontrivial. In addition, the wide range of individual miRNA concentrations in body fluids and number of clinical specimens needed to generate statistically meaningful results, require that methods selected need to have sufficient-sensitivity and dynamic range with reasonable throughputs. Commonly used miRNA measurement methods include: hybridization-based approaches (e.g., Agilent microarrays, Nanostring nCounter) [164] reverse transcription quantitative PCR arrays (RT-qPCR, e.g., TaqMan TLDA microfluidic cards, Exiqon microRNA Ready-to-Use PCR panels, *etc.*) and next generation sequencing (NGS, e.g., HiSeq 2000, SOLiD, Ion Torrent, MiSeq) [165]. Each technique presents relative strengths and weaknesses, and the choice of approach is dependent upon the research need. 

Hybridization-based methods are well established, have considerable throughput. However, array-based approaches have limited sequence specificity, sensitivity (requiring ng-μg of total input RNA), relatively limited linear and dynamic ranges (four orders of magnitude) and are difficult to use for absolute quantification and cannot identify novel miRNAs

RT-qPCR arrays are also well established and are more sensitive than hybridization-based approaches (requiring < ng-μg of total input RNA), have wider dynamic range (six orders of magnitude), and can be followed by absolute quantification on the same instrument, but with lower throughput. However, like hybridization-based approaches, prior knowledge regarding the miRNA to be measured is required, the specific assay design is non-trivial and this approach cannot be used for novel miRNA discovery.

Next generation sequencing-based methods can identify novel miRNA sequences, distinguish isomiRs, and show substantial dynamic range (five or more orders of magnitude) although, this approach requires significant starting material (ng-μg) and computational support. The analysis of multiple samples during a single run is possibly through “barcoding” in which each RNA sample is tagged with a 6-nucleotide long identifying sequence [166]. Relative quantification between datasets can be performed using a digital gene expression profiling approach [167]. A major limitation of NGS is that it is subject to critical sequence-specific bias, amplifying the detection of some miRNAs and reducing others. Systematic investigation has revealed that this bias is primarily derived from cDNA library preparation and not the NGS platform used [168,169,170,171,172]. Using a 473-member synthetic miRNA test set, researchers observed variability in miRNA abundance of up to four orders of magnitude by digital gene expression, depending on the method used to construct the library [168]. Variability in library preparation has been attributed to sequence preferences by ligation enzymes that are used to attach adapter oligonucleotides [168,170,172], differences in RNA secondary structure [173] and amplification by PCR [174,175,176]. Remarkable sequence-specific differences in product yields have been observed using both T4Rnl1 and T4Rnl2tr-catalyzed ligation. In the case of T4Rnl2tr-based adapter ligations, sequence bias was substantially alleviated by the use of a pooled mixture of 5’- and 3'- adapter sequences that varied at the two terminal nucleotides proximal to the ligation [172]. RNA secondary structure may also impact analysis by sequencing, as RNAs with 3’-stem structures are underrepresented in sequencing reads [173]. Adding further complexity to the problem, RNA may co-fold with adapter sequences, sequestering ends and making them unavailable for sequencing. PCR induced bias in NGS library construction has been observed [174] and is a function of G/C content in the sequence. Additional denaturation [177] and optimized PCR buffers [174] have been reported to reduce this bias. The use of non-amplification dependent, third generation sequencing technologies (tSMS, Helicos, Cambridge, MA) [178] may circumvent NGS limitations attributable to PCR, but these technologies are not widely available. 

### 5.6. Quantification and Validation

Lead miRNA biomarkers candidates identified through initial profiling are often directly quantified individually using RT-qPCR, an analog technology based on a fluorescent signal output relative to external standard curves [179]. This approach introduces multiple significant sources of intra- and inter-assay variation (due to differences in preparation, unequal amplification efficiency between standard and target, Monte-Carlo effect, variations in thresholding, *etc.* [179,180,181]). Digital PCR [182] is an absolute method of nucleic-acid quantification, which partitions target molecules across many replicate reactions, followed by end-point PCR. In the presence of amplification-dependent fluorescent probes, each reaction generates a highly resolved, digital output signal corresponding to the presence or absence of starting template. The starting concentration of template is then determined by Poisson statistical analysis of the number of positive and total reactions. Although this concept was introduced early in the history of PCR [183] and presents theoretical advantages over RT-qPCR based methods, practical limitations on dynamic range and throughput subsequently limited the use of dPCR. However, recent advancements in instrumentation and chemistry [184,185] circumvent these obstacles by using rapid microfluidic analysis of nano- to picoliter-sized droplet partitions (BioRad QX-200 and RainDance RainDrop, respectively), enabling the practical analysis of sizable sample sets. Droplet digital PCR (ddPCR) is highly precise, because as an absolute method of quantification it is not reliant on reference standard curves. ddPCR has recently been shown [156] to reduce analytic variability between sample preparations 37%–86% and to improve day-to-day reproducibility in the analysis of miRNAs from prostate cancer patient serum by a factor of 7 compared to RT-qPCR. As an endpoint method, ddPCR is also resistant to residual PCR inhibitors that may be present in samples [142,186], providing further robustness. 

A basic set of assay performance parameters should be included in each assay such as: limit of detection (LOD) [187], linear dynamic range [188], PCR-efficiency (where applicable) and precision. The determination of these parameters would aid in study reproducibility by eliminating spurious results based on data generated outside the operating range of the assay and provide quality assurance that the analysis is based on valid, reproducible results. The Minimum Information for Publication of Quantitative Real-Time PCR Experiments (MIQE Guidelines) provides a detailed framework for the analysis and reporting of miRNA quantification results (derived from RT-qPCR or other methods) [189]. In addition, a concise set of criteria to be reported in cell-free miRNA studies [190] has been proposed for use with MIQE:

- Sample collection: collection site, gauge and type of needle, elapsed time between collection and processing (including clotting time, if applicable), processing conditions, storage conditions
- Sample quality control: Age of sample, hemolysis measurement and cut-off criteria
- RNA isolation: sample volume, isolation kit/reagent, carrier used, spike-in used, elution/resuspension volume
- RT-qPCR: primer sequences or assay IDs, template amount, RT reagents and conditions, preamplification (if used), cDNA dilution, qPCR reagents and conditions, instrumentation, software
- Data normalization: Equations for normalization, relative quantification, standard curves, variability of endogenous controls, % recovery of spike-in, raw data

## 6. Future Prospects and Recommendations

The discovery and analysis of miRNA biomarkers from biofluids entails substantial experimental detail and complexity. Although substantial knowledge has been acquired regarding circulating miRNAs and their applications for various pathologies, their clinical diagnostic potential has yet to be realized. In part this is due to the lack of fundamental understanding of various intrinsic and extrinsic factors that may affect the spectrum of circulating RNA, but assay variability and the lack of standard protocols for miRNA measurement also play a role. To further advance the field, we will need to gain knowledge on the state of miRNA in circulation, develop commonly accepted standard operating procedures, introduce controls to assess the consistency of the results at different stages and develop proper normalization strategies. In order to improve the accuracy and reproducibility in the field, and with the aim of realizing their clinical potential, we make the following general suggestions for future studies of miRNA biomarkers from biofluids:

- To the extent that is practical, match patient with control samples as closely as possible with regard to all relevant information (age, sex, smoking status, *etc.*) except the disease status in question (e.g., cancer or benign). Minimize possible confounding variables by explicitly specifying detailed inclusion and exclusion criteria for studies and collecting specimens from donors in the most consistent manner possible (time of day, feeding status, *etc.*).
- Estimate the practical variability associated with collection (time before processing, *etc.*), storage (freezing conditions, time in freezer, *etc.*), purification (e.g., precision of extraction methods) and analysis by performing basic pilot experiments with similar specimens and report the results as supplemental materials.
- miRNAs that are highly expressed in abundant blood cells (e.g., miR-451 in RBCs, [119,138]) are likely to be derived from these cells and not from the diseased tissue, in addition to being sensitive to hemolysis, *etc.* They are therefore unlikely to be suitable as robust biomarkers and should be filtered from the data or at a minimum interpreted with caution.
- Validate measurement results using different platforms (e.g., NGS followed by RT-qPCR).
- Report the essential variables outlined in the MIQE Guidelines [189] and those recommended for cell-free miRNA studies [190].

It is also important to note that the combination of miRNA and protein-based biomarkers may provide more robust and sensitive measurement as they are susceptible to different types of errors. Despite the difficulties of getting consistent miRNA measurement results with our current technologies, we believe implementing and following a better sample preparation guideline, and new measurement technologies such as isothermal amplification will facilitate the development of miRNA based biomarkers in clinical setting.

## References

[1] Wightman B., Ha I., Ruvkun G. (1993). Posttranscriptional regulation of the heterochronic gene lin-14 by lin-4 mediates temporal pattern formation in c. Elegans. Cell.

[2] Lee R.C., Feinbaum R.L., Ambros V. (1993). The C. Elegans heterochronic gene lin-4 encodes small RNAs with antisense complementarity to lin-14. Cell.

[3] Lee R.C., Ambros V. (2001). An extensive class of small RNAs in caenorhabditis elegans. Science.

[4] Lau N.C., Lim L.P., Weinstein E.G., Bartel D.P. (2001). An abundant class of tiny RNAs with probable regulatory roles in caenorhabditis elegans. Science.

[5] Hydbring P., Badalian-Very G. (2013). Clinical applications of microRNAs. F1000Research.

[6] Lagos-Quintana M., Rauhut R., Lendeckel W., Tuschl T. (2001). Identification of novel genes coding for small expressed RNAs. Science.

[7] Ambros V. (2001). MicroRNAs: Tiny regulators with great potential. Cell.

[8] Ruvkun G. (2001). Molecular biology. Glimpses of a tiny RNA world. Science.

[9] Ruvkun G.B. (2003). The tiny RNA world. Harvey Lect..

[10] Pfeffer S., Zavolan M., Grasser F.A., Chien M., Russo J.J., Ju J., John B., Enright A.J., Marks D., Sander C. (2004). Identification of virus-encoded microRNAs. Science.

[11] Ambros V., Bartel B., Bartel D.P., Burge C.B., Carrington J.C., Chen X., Dreyfuss G., Eddy S.R., Griffiths-Jones S., Marshall M. (2003). A uniform system for microRNA annotation. RNA.

[12] Griffiths-Jones S., Saini H.K., van Dongen S., Enright A.J. (2008). Mirbase: Tools for microRNA genomics. Nucleic Acids Res..

[13] Bartel D.P. (2009). MicroRNAs: Target recognition and regulatory functions. Cell..

[14] Kim V.N. (2005). MicroRNA biogenesis: Coordinated cropping and dicing. Nat. Rev. Mol. Cell. Biol..

[15] Meister G., Landthaler M., Patkaniowska A., Dorsett Y., Teng G., Tuschl T. (2004). Human argonaute2 mediates RNA cleavage targeted by miRNAs and sirnas. Mol. Cell.

[16] Meister G., Tuschl T. (2004). Mechanisms of gene silencing by double-stranded RNA. Nature.

[17] Marti E., Pantano L., Banez-Coronel M., Llorens F., Minones-Moyano E., Porta S., Sumoy L., Ferrer I., Estivill X. (2010). A myriad of miRNA variants in control and huntington’s disease brain regions detected by massively parallel sequencing. Nucleic Acids Res..

[18] Lee L.W., Zhang S., Etheridge A., Ma L., Martin D., Galas D., Wang K. (2010). Complexity of the microRNA repertoire revealed by next-generation sequencing. RNA.

[19] Guduric-Fuchs J., O’Connor A., Cullen A., Harwood L., Medina R.J., O’Neill C.L., Stitt A.W., Curtis T.M., Simpson D.A. (2012). Deep sequencing reveals predominant expression of mir-21 amongst the small non-coding RNAs in retinal microvascular endothelial cells. J. Cell. Biochem..

[20] Voellenkle C., Rooij J., Guffanti A., Brini E., Fasanaro P., Isaia E., Croft L., David M., Capogrossi M.C., Moles A. (2012). Deep-sequencing of endothelial cells exposed to hypoxia reveals the complexity of known and novel microRNAs. RNA.

[21] Cloonan N., Wani S., Xu Q., Gu J., Lea K., Heater S., Barbacioru C., Steptoe A.L., Martin H.C., Nourbakhsh E. (2011). MicroRNAs and their isomirs function cooperatively to target common biological pathways. Genome Biol..

[22] Li S.C., Liao Y.L., Ho M.R., Tsai K.W., Lai C.H., Lin W.C. (2012). miRNA arm selection and isomir distribution in gastric cancer. BMC Genomics.

[23] Zhou H., Arcila M.L., Li Z., Lee E.J., Henzler C., Liu J., Rana T.M., Kosik K.S. (2012). Deep annotation of mouse iso-mir and iso-mor variation. Nucleic Acids Res..

[24] Guo H., Ingolia N.T., Weissman J.S., Bartel D.P. (2010). Mammalian microRNAs predominantly act to decrease target mRNA levels. Nature.

[25] Kloosterman W.P., Plasterk R.H. (2006). The diverse functions of microRNAs in animal development and disease. Dev. Cell..

[26] Stefani G., Slack F.J. (2008). Small non-coding RNAs in animal development. Nat. Rev. Mol. Cell. Biol.

[27] Friedman R.C., Farh K.K., Burge C.B., Bartel D.P. (2009). Most mammalian mRNAs are conserved targets of microRNAs. Genome Res..

[28] Helwak A., Kudla G., Dudnakova T., Tollervey D. (2013). Mapping the human miRNA interactome by clash reveals frequent noncanonical binding. Cell.

[29] Landgraf P., Rusu M., Sheridan R., Sewer A., Iovino N., Aravin A., Pfeffer S., Rice A., Kamphorst A.O., Landthaler M. (2007). A mammalian microRNA expression atlas based on small RNA library sequencing. Cell.

[30] Esquela-Kerscher A., Slack F.J. (2006). Oncomirs - microRNAs with a role in cancer. Nat. Rev. Cancer.

[31] Calin G.A., Liu C.G., Sevignani C., Ferracin M., Felli N., Dumitru C.D., Shimizu M., Cimmino A., Zupo S., Dono M. (2004). MicroRNA profiling reveals distinct signatures in b cell chronic lymphocytic leukemias. Proc. Natl. Acad. Sci. USA.

[32] Lu J., Getz G., Miska E.A., Alvarez-Saavedra E., Lamb J., Peck D., Sweet-Cordero A., Ebert B.L., Mak R.H., Ferrando A.A. (2005). MicroRNA expression profiles classify human cancers. Nature.

[33] Porkka K.P., Pfeiffer M.J., Waltering K.K., Vessella R.L., Tammela T.L., Visakorpi T. (2007). MicroRNA expression profiling in prostate cancer. Cancer Res..

[34] Albinsson S., Suarez Y., Skoura A., Offermanns S., Miano J.M., Sessa W.C. (2010). MicroRNAs are necessary for vascular smooth muscle growth, differentiation, and function. Arterioscler. Thromb. Vasc. Biol..

[35] Zhao Y., Ransom J.F., Li A., Vedantham V., von Drehle M., Muth A.N., Tsuchihashi T., McManus M.T., Schwartz R.J., Srivastava D. (2007). Dysregulation of cardiogenesis, cardiac conduction, and cell cycle in mice lacking miRNA-1–2. Cell.

[36] Yang B., Lin H., Xiao J., Lu Y., Luo X., Li B., Zhang Y., Xu C., Bai Y., Wang H. (2007). The muscle-specific microRNA mir-1 regulates cardiac arrhythmogenic potential by targeting gja1 and kcnj2. Nat. Med..

[37] Van Rooij E., Sutherland L.B., Liu N., Williams A.H., McAnally J., Gerard R.D., Richardson J.A., Olson E.N. (2006). A signature pattern of stress-responsive microRNAs that can evoke cardiac hypertrophy and heart failure. Proc. Natl. Acad. Sci. USA.

[38] Fang Y., Shi C., Manduchi E., Civelek M., Davies P.F. (2010). MicroRNA-10a regulation of proinflammatory phenotype in athero-susceptible endothelium *in vivo* and *in vitro*. Proc. Natl. Acad. Sci. USA.

[39] Cordes K.R., Sheehy N.T., White M.P., Berry E.C., Morton S.U., Muth A.N., Lee T.H., Miano J.M., Ivey K.N., Srivastava D. (2009). Mir-145 and mir-143 regulate smooth muscle cell fate and plasticity. Nature.

[40] Nicoli S., Standley C., Walker P., Hurlstone A., Fogarty K.E., Lawson N.D. (2010). MicroRNA-mediated integration of haemodynamics and vegf signalling during angiogenesis. Nature.

[41] Esteller M. (2011). Non-coding RNAs in human disease. Nat. Rev. Genet..

[42] Gehrke S., Imai Y., Sokol N., Lu B. (2010). Pathogenic lrrk2 negatively regulates microRNA-mediated translational repression. Nature.

[43] Haramati S., Chapnik E., Sztainberg Y., Eilam R., Zwang R., Gershoni N., McGlinn E., Heiser P.W., Wills A.M., Wirguin I. (2010). MiRNA malfunction causes spinal motor neuron disease. Proc. Natl. Acad. Sci. USA.

[44] Lee Y., Samaco R.C., Gatchel J.R., Thaller C., Orr H.T., Zoghbi H.Y. (2008). Mir-19, mir-101 and mir-130 co-regulate atxn1 levels to potentially modulate sca1 pathogenesis. Nat. Neurosci..

[45] Hebert S.S., Horre K., Nicolai L., Papadopoulou A.S., Mandemakers W., Silahtaroglu A.N., Kauppinen S., Delacourte A., de Strooper B. (2008). Loss of microRNA cluster mir-29a/b-1 in sporadic alzheimer’s disease correlates with increased bace1/beta-secretase expression. Proc. Natl. Acad. Sci. USA.

[46] Wang W.X., Rajeev B.W., Stromberg A.J., Ren N., Tang G., Huang Q., Rigoutsos I., Nelson P.T. (2008). The expression of microRNA mir-107 decreases early in alzheimer’s disease and may accelerate disease progression through regulation of beta-site amyloid precursor protein-cleaving enzyme 1. J. Neurosci..

[47] Volinia S., Calin G.A., Liu C.G., Ambs S., Cimmino A., Petrocca F., Visone R., Iorio M., Roldo C., Ferracin M. (2006). A microRNA expression signature of human solid tumors defines cancer gene targets. Proc. Natl. Acad. Sci. USA.

[48] Blenkiron C., Goldstein L.D., Thorne N.P., Spiteri I., Chin S.F., Dunning M.J., Barbosa-Morais N.L., Teschendorff A.E., Green A.R., Ellis I.O. (2007). MicroRNA expression profiling of human breast cancer identifies new markers of tumor subtype. Genome Biol..

[49] Sempere L.F., Christensen M., Silahtaroglu A., Bak M., Heath C.V., Schwartz G., Wells W., Kauppinen S., Cole C.N. (2007). Altered microRNA expression confined to specific epithelial cell subpopulations in breast cancer. Cancer Res..

[50] Mandel P., Metais P. (1948). Les acides nucléiques du plasma sanguin chez l’homme. CR Acad. Sci. Paris.

[51] Bendich A., Wilczok T., Borenfreund E. (1965). Circulating DNA as a possible factor in oncogenesis. Science.

[52] Tan E.M., Schur P.H., Carr R.I., Kunkel H.G. (1966). Deoxybonucleic acid (DNA) and antibodies to DNA in the serum of patients with systemic lupus erythematosus. J. Clin Invest..

[53] Kamm R.C., Smith A.G. (1972). Nucleic acid concentrations in normal human plasma. Clin. Chem..

[54] Stroun M., Anker P., Maurice P., Gahan P.B. (1977). Circulating nucleic acids in higher organisms. Int. Rev. Cytol..

[55] El-Hefnawy T., Raja S., Kelly L., Bigbee W.L., Kirkwood J.M., Luketich J.D., Godfrey T.E. (2004). Characterization of amplifiable, circulating RNA in plasma and its potential as a tool for cancer diagnostics. Clin. Chem..

[56] Chen X.Q., Bonnefoi H., Pelte M.F., Lyautey J., Lederrey C., Movarekhi S., Schaeffer P., Mulcahy H.E., Meyer P., Stroun M. (2000). Telomerase RNA as a detection marker in the serum of breast cancer patients. Clin. Cancer Res..

[57] Hasselmann D.O., Rappl G., Rossler M., Ugurel S., Tilgen W., Reinhold U. (2001). Detection of tumor-associated circulating mRNA in serum, plasma and blood cells from patients with disseminated malignant melanoma. Oncol. Rep..

[58] Anker P., Lefort F., Vasioukhin V., Lyautey J., Lederrey C., Chen X.Q., Stroun M., Mulcahy H.E., Farthing M.J. (1997). K-ras mutations are found in DNA extracted from the plasma of patients with colorectal cancer. Gastroenterology.

[59] Kamm R.C., Smith A.G. (1972). Ribonuclease activity in human plasma. Clin. Biochem..

[60] Xi Y., Nakajima G., Gavin E., Morris C.G., Kudo K., Hayashi K., Ju J. (2007). Systematic analysis of microRNA expression of RNA extracted from fresh frozen and formalin-fixed paraffin-embedded samples. RNA.

[61] Lawrie C.H., Gal S., Dunlop H.M., Pushkaran B., Liggins A.P., Pulford K., Banham A.H., Pezzella F., Boultwood J., Wainscoat J.S. (2008). Detection of elevated levels of tumour-associated microRNAs in serum of patients with diffuse large b-cell lymphoma. Br. J. Haematol..

[62] Mitchell P.S., Parkin R.K., Kroh E.M., Fritz B.R., Wyman S.K., Pogosova-Agadjanyan E.L., Peterson A., Noteboom J., O’Briant K.C., Allen A. (2008). Circulating microRNAs as stable blood-based markers for cancer detection. Proc. Natl. Acad. Sci. USA.

[63] Roth C., Rack B., Muller V., Janni W., Pantel K., Schwarzenbach H. (2010). Circulating microRNAs as blood-based markers for patients with primary and metastatic breast cancer. Breast Cancer Res..

[64] Ng E.K., Chong W.W., Jin H., Lam E.K., Shin V.Y., Yu J., Poon T.C., Ng S.S., Sung J.J. (2009). Differential expression of microRNAs in plasma of patients with colorectal cancer: A potential marker for colorectal cancer screening. Gut.

[65] Asaga S., Kuo C., Nguyen T., Terpenning M., Giuliano A.E., Hoon D.S. (2011). Direct serum assay for microRNA-21 concentrations in early and advanced breast cancer. Clin. Chem..

[66] Hu Z., Chen X., Zhao Y., Tian T., Jin G., Shu Y., Chen Y., Xu L., Zen K., Zhang C. (2010). Serum microRNA signatures identified in a genome-wide serum microRNA expression profiling predict survival of non-small-cell lung cancer. J. Clin. Oncol..

[67] Schwarzenbach H., Hoon D.S., Pantel K. (2011). Cell-free nucleic acids as biomarkers in cancer patients. Nat. Rev. Cancer.

[68] Wang J., Zhang K.Y., Liu S.M., Sen S. (2014). Tumor-associated circulating microRNAs as biomarkers of cancer. Molecules.

[69] Wang K., Zhang S., Marzolf B., Troisch P., Brightman A., Hu Z., Hood L.E., Galas D.J. (2009). Circulating microRNAs, potential biomarkers for drug-induced liver injury. Proc. Natl. Acad. Sci. USA.

[70] Hu Z., Lausted C., Yoo H., Yan X., Brightman A., Chen J., Wang W., Bu X., Hood L. (2014). Quantitative liver-specific protein fingerprint in blood: A signature for hepatotoxicity. Theranostics.

[71] Fichtlscherer S., de Rosa S., Fox H., Schwietz T., Fischer A., Liebetrau C., Weber M., Hamm C.W., Roxe T., Muller-Ardogan M. (2010). Circulating microRNAs in patients with coronary artery disease. Circ. Res..

[72] Wang G.K., Zhu J.Q., Zhang J.T., Li Q., Li Y., He J., Qin Y.W., Jing Q. (2010). Circulating microRNA: A novel potential biomarker for early diagnosis of acute myocardial infarction in humans. Eur. Heart J..

[73] Adachi T., Nakanishi M., Otsuka Y., Nishimura K., Hirokawa G., Goto Y., Nonogi H., Iwai N. (2010). Plasma microRNA 499 as a biomarker of acute myocardial infarction. Clin. Chem..

[74] Li C., Fang Z., Jiang T., Zhang Q., Liu C., Zhang C., Xiang Y. (2013). Serum microRNAs profile from genome-wide serves as a fingerprint for diagnosis of acute myocardial infarction and angina pectoris. BMC Med. Genomics.

[75] Schipper H.M., Maes O.C., Chertkow H.M., Wang E. (2007). MicroRNA expression in alzheimer blood mononuclear cells. Gene Regul. Syst. Bio..

[76] Cogswell J.P., Ward J., Taylor I.A., Waters M., Shi Y., Cannon B., Kelnar K., Kemppainen J., Brown D., Chen C. (2008). Identification of miRNA changes in alzheimer’s disease brain and csf yields putative biomarkers and insights into disease pathways. J. Alzheimers Dis..

[77] Alexandrov P.N., Dua P., Hill J.M., Bhattacharjee S., Zhao Y., Lukiw W.J. (2012). MicroRNA (miRNA) speciation in alzheimer’s disease (ad) cerebrospinal fluid (csf) and extracellular fluid (ecf). Int. J. Biochem. Mol. Biol..

[78] Geekiyanage H., Jicha G.A., Nelson P.T., Chan C. (2012). Blood serum miRNA: Non-invasive biomarkers for alzheimer’s disease. Exp. Neurol..

[79] Villa C., Ridolfi E., Fenoglio C., Ghezzi L., Vimercati R., Clerici F., Marcone A., Gallone S., Serpente M., Cantoni C. (2013). Expression of the transcription factor sp1 and its regulatory hsa-mir-29b in peripheral blood mononuclear cells from patients with alzheimer’s disease. J. Alzheimers Dis..

[80] Bekris L.M., Lutz F., Montine T.J., Yu C.E., Tsuang D., Peskind E.R., Leverenz J.B. (2013). MicroRNA in alzheimer’s disease: An exploratory study in brain, cerebrospinal fluid and plasma. Biomarkers.

[81] Kumar P., Dezso Z., MacKenzie C., Oestreicher J., Agoulnik S., Byrne M., Bernier F., Yanagimachi M., Aoshima K., Oda Y. (2013). Circulating miRNA biomarkers for alzheimer’s disease. PLoS One.

[82] Bhatnagar S., Chertkow H., Schipper H.M., Yuan Z., Shetty V., Jenkins S., Jones T., Wang E. (2014). Increased microRNA-34c abundance in alzheimer’s disease circulating blood plasma. Front. Mol. Neurosci..

[83] Muller M., Kuiperij H.B., Claassen J.A., Kusters B., Verbeek M.M. (2014). MicroRNAs in alzheimer’s disease: Differential expression in hippocampus and cell-free cerebrospinal fluid. Neurobiol. Aging.

[84] Margis R., Margis R., Rieder C.R. (2011). Identification of blood microRNAs associated to parkinsonis disease. J. Biotechnol..

[85] Martins M., Rosa A., Guedes L.C., Fonseca B.V., Gotovac K., Violante S., Mestre T., Coelho M., Rosa M.M., Martin E.R. (2011). Convergence of miRNA expression profiling, alpha-synuclein interacton and gwas in parkinson’s disease. PLoS One.

[86] Khoo S.K., Petillo D., Kang U.J., Resau J.H., Berryhill B., Linder J., Forsgren L., Neuman L.A., Tan A.C. (2012). Plasma-based circulating microRNA biomarkers for parkinson’s disease. J. Parkinsons Dis..

[87] Cardo L.F., Coto E., de Mena L., Ribacoba R., Moris G., Menendez M., Alvarez V. (2013). Profile of microRNAs in the plasma of parkinson’s disease patients and healthy controls. J. Neurol..

[88] Soreq L., Salomonis N., Bronstein M., Greenberg D.S., Israel Z., Bergman H., Soreq H. (2013). Small RNA sequencing-microarray analyses in parkinson leukocytes reveal deep brain stimulation-induced splicing changes that classify brain region transcriptomes. Front. Mol. Neurosci..

[89] Gaughwin P.M., Ciesla M., Lahiri N., Tabrizi S.J., Brundin P., Bjorkqvist M. (2011). Hsa-mir-34b is a plasma-stable microRNA that is elevated in pre-manifest huntington’s disease. Hum. Mol. Genet..

[90] Hoekstra M., van der Lans C.A., Halvorsen B., Gullestad L., Kuiper J., Aukrust P., van Berkel T.J., Biessen E.A. (2010). The peripheral blood mononuclear cell microRNA signature of coronary artery disease. Biochem. Biophys. Res. Commun..

[91] Takahashi Y., Satoh M., Minami Y., Tabuchi T., Itoh T., Nakamura M. (2010). Expression of mir-146a/b is associated with the toll-like receptor 4 signal in coronary artery disease: Effect of renin-angiotensin system blockade and statins on miRNA-146a/b and toll-like receptor 4 levels. Clin. Sci..

[92] Weber M., Baker M.B., Patel R.S., Quyyumi A.A., Bao G., Searles C.D. (2011). microRNA expression profile in cad patients and the impact of acei/arb. Cardiol. Res. Pract..

[93] Lu H.Q., Liang C., He Z.Q., Fan M., Wu Z.G. (2013). Circulating mir-214 is associated with the severity of coronary artery disease. J. Geriatr. Cardiol..

[94] Ai J., Zhang R., Li Y., Pu J., Lu Y., Jiao J., Li K., Yu B., Li Z., Wang R. (2010). Circulating microRNA-1 as a potential novel biomarker for acute myocardial infarction. Biochem. Biophys. Res. Commun..

[95] Cheng Y., Tan N., Yang J., Liu X., Cao X., He P., Dong X., Qin S., Zhang C. (2010). A translational study of circulating cell-free microRNA-1 in acute myocardial infarction. Clin. Sci..

[96] Bonora E., Kiechl S., Willeit J., Oberhollenzer F., Egger G., Meigs J.B., Bonadonna R.C., Muggeo M., Bruneck S. (2004). Population-based incidence rates and risk factors for type 2 diabetes in white individuals: The bruneck study. Diabetes.

[97] Zampetaki A., Willeit P., Tilling L., Drozdov I., Prokopi M., Renard J.M., Mayr A., Weger S., Schett G., Shah A. (2012). Prospective study on circulating microRNAs and risk of myocardial infarction. J. Am. Coll. Cardiol..

[98] Vogel B., Keller A., Frese K.S., Kloos W., Kayvanpour E., Sedaghat-Hamedani F., Hassel S., Marquart S., Beier M., Giannitis E. (2013). Refining diagnostic microRNA signatures by whole-mirnome kinetic analysis in acute myocardial infarction. Clin. Chem..

[99] Wang F., Long G., Zhao C., Li H., Chaugai S., Wang Y., Chen C., Wang D.W. (2013). Plasma microRNA-133a is a new marker for both acute myocardial infarction and underlying coronary artery stenosis. J. Transl. Med..

[100] Endo K., Naito Y., Ji X., Nakanishi M., Noguchi T., Goto Y., Nonogi H., Ma X., Weng H., Hirokawa G. (2013). MicroRNA 210 as a biomarker for congestive heart failure. Biol. Pharm. Bull..

[101] Fukushima Y., Nakanishi M., Nonogi H., Goto Y., Iwai N. (2011). Assessment of plasma miRNAs in congestive heart failure. Circ. J..

[102] Kin K., Miyagawa S., Fukushima S., Shirakawa Y., Torikai K., Shimamura K., Daimon T., Kawahara Y., Kuratani T., Sawa Y. (2012). Tissue- and plasma-specific microRNA signatures for atherosclerotic abdominal aortic aneurysm. J. Am. Heart Assoc..

[103] Sepramaniam S., Tan J.R., Tan K.S., DeSilva D.A., Tavintharan S., Woon F.P., Wang C.W., Yong F.L., Karolina D.S., Kaur P. (2014). Circulating microRNAs as biomarkers of acute stroke. Int. J. Mol. Sci..

[104] Gan C.S., Wang C.W., Tan K.S. (2012). Circulatory microRNA-145 expression is increased in cerebral ischemia. Genet. Mol. Res..

[105] Li T., Cao H., Zhuang J., Wan J., Guan M., Yu B., Li X., Zhang W. (2011). Identification of mir-130a, mir-27b and mir-210 as serum biomarkers for atherosclerosis obliterans. Clin. Chim. Acta.

[106] Nielsen L.B., Wang C., Sorensen K., Bang-Berthelsen C.H., Hansen L., Andersen M.L., Hougaard P., Juul A., Zhang C.Y., Pociot F. (2012). Circulating levels of microRNA from children with newly diagnosed type 1 diabetes and healthy controls: Evidence that mir-25 associates to residual beta-cell function and glycaemic control during disease progression. Exp. Diabetes Res..

[107] Zampetaki A., Kiechl S., Drozdov I., Willeit P., Mayr U., Prokopi M., Mayr A., Weger S., Oberhollenzer F., Bonora E. (2010). Plasma microRNA profiling reveals loss of endothelial mir-126 and other microRNAs in type 2 diabetes. Circ. Res..

[108] Kong L., Zhu J., Han W., Jiang X., Xu M., Zhao Y., Dong Q., Pang Z., Guan Q., Gao L. (2011). Significance of serum microRNAs in pre-diabetes and newly diagnosed type 2 diabetes: A clinical study. Acta Diabetol..

[109] Rong Y., Bao W., Shan Z., Liu J., Yu X., Xia S., Gao H., Wang X., Yao P., Hu F.B. (2013). Increased microRNA-146a levels in plasma of patients with newly diagnosed type 2 diabetes mellitus. PLoS One.

[110] Peng H., Zhong M., Zhao W., Wang C., Zhang J., Liu X., Li Y., Paudel S.D., Wang Q., Lou T. (2013). Urinary mir-29 correlates with albuminuria and carotid intima-media thickness in type 2 diabetes patients. PLoS One.

[111] Wang X., Sundquist J., Zoller B., Memon A.A., Palmer K., Sundquist K., Bennet L. (2014). Determination of 14 circulating microRNAs in swedes and iraqis with and without diabetes mellitus type 2. PLoS One.

[112] Zhao C., Dong J., Jiang T., Shi Z., Yu B., Zhu Y., Chen D., Xu J., Huo R., Dai J. (2011). Early second-trimester serum miRNA profiling predicts gestational diabetes mellitus. PLoS One.

[113] Pan B.T., Teng K., Wu C., Adam M., Johnstone R.M. (1985). Electron microscopic evidence for externalization of the transferrin receptor in vesicular form in sheep reticulocytes. J. Cell Biol..

[114] Harding C., Heuser J., Stahl P. (1984). Endocytosis and intracellular processing of transferrin and colloidal gold-transferrin in rat reticulocytes: Demonstration of a pathway for receptor shedding. Eur. J. Cell Biol..

[115] Raposo G., Stoorvogel W. (2013). Extracellular vesicles: Exosomes, microvesicles, and friends. J. Cell Biol..

[116] Valadi H., Ekstrom K., Bossios A., Sjostrand M., Lee J.J., Lotvall J.O. (2007). Exosome-mediated transfer of mRNAs and microRNAs is a novel mechanism of genetic exchange between cells. Nat. Cell. Biol..

[117] Weber J.A., Baxter D.H., Zhang S., Huang D.Y., Huang K.H., Lee M.J., Galas D.J., Wang K. (2010). The microRNA spectrum in 12 body fluids. Clin. Chem..

[118] Morello M., Minciacchi V.R., de Candia P., Yang J., Posadas E., Kim H., Griffiths D., Bhowmick N., Chung L.W., Gandellini P. (2013). Large oncosomes mediate intercellular transfer of functional microRNA. Cell Cycle.

[119] Pritchard C.C., Kroh E., Wood B., Arroyo J.D., Dougherty K.J., Miyaji M.M., Tait J.F., Tewari M. (2012). Blood cell origin of circulating microRNAs: A cautionary note for cancer biomarker studies. Cancer Prev. Res. (Phila).

[120] Cheng H.H., Yi H.S., Kim Y., Kroh E.M., Chien J.W., Eaton K.D., Goodman M.T., Tait J.F., Tewari M., Pritchard C.C. (2013). Plasma processing conditions substantially influence circulating microRNA biomarker levels. PLoS One.

[121] Nilsson R.J., Balaj L., Hulleman E., van Rijn S., Pegtel D.M., Walraven M., Widmark A., Gerritsen W.R., Verheul H.M., Vandertop W.P. (2011). Blood platelets contain tumor-derived RNA biomarkers. Blood.

[122] Wang Z.P., Eisenberger M.A., Carducci M.A., Partin A.W., Scher H.I., Ts’o P.O. (2000). Identification and characterization of circulating prostate carcinoma cells. Cancer.

[123] Skog J., Wurdinger T., van Rijn S., Meijer D.H., Gainche L., Sena-Esteves M., Curry W.T., Carter B.S., Krichevsky A.M., Breakefield X.O. (2008). Glioblastoma microvesicles transport RNA and proteins that promote tumour growth and provide diagnostic biomarkers. Nat. Cell. Biol..

[124] Stoorvogel W. (2012). Functional transfer of microRNA by exosomes. Blood.

[125] Montecalvo A., Larregina A.T., Shufesky W.J., Stolz D.B., Sullivan M.L., Karlsson J.M., Baty C.J., Gibson G.A., Erdos G., Wang Z. (2012). Mechanism of transfer of functional microRNAs between mouse dendritic cells via exosomes. Blood.

[126] Kharaziha P., Ceder S., Li Q., Panaretakis T. (2012). Tumor cell-derived exosomes: A message in a bottle. Biochim Biophys Acta.

[127] Arroyo J.D., Chevillet J.R., Kroh E.M., Ruf I.K., Pritchard C.C., Gibson D.F., Mitchell P.S., Bennett C.F., Pogosova-Agadjanyan E.L., Stirewalt D.L. (2011). Argonaute2 complexes carry a population of circulating microRNAs independent of vesicles in human plasma. Proc. Natl. Acad. Sci. USA.

[128] Turchinovich A., Weiz L., Langheinz A., Burwinkel B. (2011). Characterization of extracellular circulating microRNA. Nucl. Acid. Res..

[129] Vickers K.C., Palmisano B.T., Shoucri B.M., Shamburek R.D., Remaley A.T. (2011). MicroRNAs are transported in plasma and delivered to recipient cells by high-density lipoproteins. Nat. Cell. Biol..

[130] Wang K., Zhang S., Weber J., Baxter D., Galas D.J. (2010). Export of microRNAs and microRNA-protective protein by mammalian cells. Nucl. Acid. Res..

[131] Wang K., Li H., Yuan Y., Etheridge A., Zhou Y., Huang D., Wilmes P., Galas D. (2012). The complex exogenous RNA spectra in human plasma: An interface with human gut biota?. PLoS One.

[132] Semenov D.V., Baryakin D.N., Brenner E.V., Kurilshikov A.M., Vasiliev G.V., Bryzgalov L.A., Chikova E.D., Filippova J.A., Kuligina E.V., Richter V.A. (2012). Unbiased approach to profile the variety of small non-coding RNA of human blood plasma with massively parallel sequencing technology. Expert Opin. Biol. Ther..

[133] Timmons L., Fire A. (1998). Specific interference by ingested dsRNA. Nature.

[134] Macron D. (2009). Rosetta genomics says ‘technical obstacles’ will delay colorectal cancer screen launch. GenomeWeb.

[135] National Institutes of Health Extracellular RNA communication. https://commonfund.nih.gov/Exrna/overview.

[136] Wang K., Yuan Y., Cho J.H., McClarty S., Baxter D., Galas D.J. (2012). Comparing the microRNA spectrum between serum and plasma. PLoS One.

[137] Dhingra N., Diepart M., Dziekan G., Khamassi S., Otaiza F., Wilburn S. (2010). WHO Guidelines on Drawing Blood: Best Practices in Phlebotomy.

[138] Kirschner M.B., Edelman J.J., Kao S.C., Vallely M.P., van Zandwijk N., Reid G. (2013). The impact of hemolysis on cell-free microRNA biomarkers. Front. Genet..

[139] McDonald J.S., Milosevic D., Reddi H.V., Grebe S.K., Algeciras-Schimnich A. (2011). Analysis of circulating microRNA: Preanalytical and analytical challenges. Clin. Chem..

[140] Vaught J.B. (2006). Blood collection, shipment, processing, and storage. Cancer Epidemiol. Biomark. Prev..

[141] Liumbruno G.M., Catalano L., Piccinini V., Pupella S., Grazzini G. (2009). Reduction of the risk of bacterial contamination of blood components through diversion of the first part of the donation of blood and blood components. Blood Trans..

[142] Kim D.J., Linnstaedt S., Palma J., Park J.C., Ntrivalas E., Kwak-Kim J.Y., Gilman-Sachs A., Beaman K., Hastings M.L., Martin J.N. (2012). Plasma components affect accuracy of circulating cancer-related microRNA quantitation. J. Mol. Diagn..

[143] Johnson M.L., Navanukraw C., Grazul-Bilska A.T., Reynolds L.P., Redmer D.A. (2003). Heparinase treatment of RNA before quantitative real-time rt-pcr. Biotechniques.

[144] Hastings M.L., Palma J., Duelli D.M. (2012). Sensitive pcr-based quantitation of cell-free circulating microRNAs. Methods.

[145] Duttagupta R., Jiang R., Gollub J., Getts R.C., Jones K.W. (2011). Impact of cellular miRNAs on circulating miRNA biomarker signatures. PLoS One.

[146] Kroh E.M., Parkin R.K., Mitchell P.S., Tewari M. (2010). Analysis of circulating microRNA biomarkers in plasma and serum using quantitative reverse transcription-PCR (QRT-PCR). Methods.

[147] Page K., Guttery D.S., Zahra N., Primrose L., Elshaw S.R., Pringle J.H., Blighe K., Marchese S.D., Hills A., Woodley L. (2013). Influence of plasma processing on recovery and analysis of circulating nucleic acids. PLoS One.

[148] Andreasen D., Fog J.U., Biggs W., Salomon J., Dahslveen I.K., Baker A., Mouritzen P. (2010). Improved microRNA quantification in total RNA from clinical samples. Methods.

[149] Tauro B.J., Greening D.W., Mathias R.A., Ji H., Mathivanan S., Scott A.M., Simpson R.J. (2012). Comparison of ultracentrifugation, density gradient separation, and immunoaffinity capture methods for isolating human colon cancer cell line lim1863-derived exosomes. Methods.

[150] Thery C., Amigorena S., Raposo G., Clayton A. (2006). Isolation and characterization of exosomes from cell culture supernatants and biological fluids. Curr. Protoc. Cell Biol..

[151] Lasser C., Eldh M., Lotvall J. (2012). Isolation and characterization of RNA-containing exosomes. J. Vis. Exp..

[152] Quackenbush J.F., Cassidy P.B., Pfeffer L.M., Boucher K.M., Hawkes J.E., Pfeffer S.R., Kopelovich L., Leachman S.A. (2014). Isolation of circulating microRNAs from microvesicles found in human plasma. Methods Mol. Biol..

[153] Sohel M.M., Hoelker M., Noferesti S.S., Salilew-Wondim D., Tholen E., Looft C., Rings F., Uddin M.J., Spencer T.E., Schellander K. (2013). Exosomal and non-exosomal transport of extra-cellular microRNAs in follicular fluid: Implications for bovine oocyte developmental competence. PLoS One.

[154] Witwer K.W., Buzas E.I., Bemis L.T., Bora A., Lasser C., Lotvall J., Nolte-'t Hoen E.N., Piper M.G., Sivaraman S., Skog J. (2013). Standardization of sample collection, isolation and analysis methods in extracellular vesicle research. J. Extracell. Vesicles..

[155] Blondal T., Jensby Nielsen S., Baker A., Andreasen D., Mouritzen P., Wrang Teilum M., Dahlsveen I.K. (2013). Assessing sample and miRNA profile quality in serum and plasma or other biofluids. Methods.

[156] Hindson C.M., Chevillet J.R., Briggs H.A., Gallichotte E.N., Ruf I.K., Hindson B.J., Vessella R.L., Tewari M. (2013). Absolute quantification by droplet digital pcr *versus* analog real-time PCR. Nat. Methods.

[157] Delviks-Frankenberry K., Cingoz O., Coffin J.M., Pathak V.K. (2012). Recombinant origin, contamination, and de-discovery of xmrv. Curr. Opin. Virol..

[158] Alter H.J., Mikovits J.A., Switzer W.M., Ruscetti F.W., Lo S.C., Klimas N., Komaroff A.L., Montoya J.G., Bateman L., Levine S. A multicenter blinded analysis indicates no association between chronic fatigue syndrome/myalgic encephalomyelitis and either xenotropic murine leukemia virus-related virus or polytropic murine leukemia virus. MBio.

[159] Zheng H., Jia H., Shankar A., Heneine W., Switzer W.M. (2011). Detection of murine leukemia virus or mouse DNA in commercial rt-pcr reagents and human dnas. PLoS One.

[160] Lo Y.M., Chan K.C. (2006). Setting up a polymerase chain reaction laboratory. Methods Mol. Biol..

[161] Dieffenbach C.W., Dveksler G.S. (1993). Setting up a PCR laboratory. PCR Meth. Appl..

[162] Pritchard C.C., Cheng H.H., Tewari M. (2012). MicroRNA profiling: Approaches and considerations. Nat. Rev. Genet..

[163] Baker M. (2010). MicroRNA profiling: Separating signal from noise. Nat. Methods.

[164] Geiss G.K., Bumgarner R.E., Birditt B., Dahl T., Dowidar N., Dunaway D.L., Fell H.P., Ferree S., George R.D., Grogan T. (2008). Direct multiplexed measurement of gene expression with color-coded probe pairs. Nat. Biotechnol..

[165] Metzker M.L. (2010). Sequencing technologies - the next generation. Nat. Rev. Genet..

[166] Smith A.M., Heisler L.E., St Onge R.P., Farias-Hesson E., Wallace I.M., Bodeau J., Harris A.N., Perry K.M., Giaever G., Pourmand N. (2010). Highly-multiplexed barcode sequencing: An efficient method for parallel analysis of pooled samples. Nucl. Acid. Res..

[167] Kuchenbauer F., Morin R.D., Argiropoulos B., Petriv O.I., Griffith M., Heuser M., Yung E., Piper J., Delaney A., Prabhu A.L. (2008). In-depth characterization of the microRNA transcriptome in a leukemia progression model. Genome Res..

[168] Linsen S.E., de Wit E., Janssens G., Heater S., Chapman L., Parkin R.K., Fritz B., Wyman S.K., de Bruijn E., Voest E.E. (2009). Limitations and possibilities of small RNA digital gene expression profiling. Nat. Methods.

[169] Tian G., Yin X., Luo H., Xu X., Bolund L., Zhang X., Gan S.Q., Li N. (2010). Sequencing bias: Comparison of different protocols of microRNA library construction. BMC Biotechnol..

[170] Hafner M., Renwick N., Brown M., Mihailovic A., Holoch D., Lin C., Pena J.T., Nusbaum J.D., Morozov P., Ludwig J. (2011). RNA-ligase-dependent biases in miRNA representation in deep-sequenced small RNA cdna libraries. RNA.

[171] Raabe C.A., Tang T.H., Brosius J., Rozhdestvensky T.S. (2014). Biases in small RNA deep sequencing data. Nucl. Acid. Res..

[172] Jayaprakash A.D., Jabado O., Brown B.D., Sachidanandam R. (2011). Identification and remediation of biases in the activity of RNA ligases in small-rna deep sequencing. Nucl. Acid. Res..

[173] Zhuang F., Fuchs R.T., Sun Z., Zheng Y., Robb G.B. (2012). Structural bias in t4 RNA ligase-mediated 3'-adapter ligation. Nucl. Acid. Res..

[174] Dabney J., Meyer M. (2012). Length and gc-biases during sequencing library amplification: A comparison of various polymerase-buffer systems with ancient and modern DNA sequencing libraries. Biotechniques.

[175] Orpana A.K., Ho T.H., Stenman J. (2012). Multiple heat pulses during pcr extension enabling amplification of gc-rich sequences and reducing amplification bias. Anal. Chem..

[176] Sendler E., Johnson G.D., Krawetz S.A. (2011). Local and global factors affecting RNA sequencing analysis. Anal. Biochem..

[177] Aird D., Ross M.G., Chen W.S., Danielsson M., Fennell T., Russ C., Jaffe D.B., Nusbaum C., Gnirke A. (2011). Analyzing and minimizing PCR amplification bias in illumina sequencing libraries. Genome Biol..

[178] Kapranov P., Ozsolak F., Milos P.M. (2012). Profiling of short RNAs using helicos single-molecule sequencing. Methods Mol. Biol..

[179] Nolan T., Hands R.E., Bustin S.A. (2006). Quantification of mRNA using real-time RT-PCR. Nat. Protoc..

[180] Karlen Y., McNair A., Perseguers S., Mazza C., Mermod N. (2007). Statistical significance of quantitative PCR. BMC Bioinf..

[181] Bustin S.A., Nolan T. (2004). Pitfalls of quantitative real-time reverse-transcription polymerase chain reaction. J. Biomol. Tech..

[182] Vogelstein B., Kinzler K.W. (1999). Digital PCR. Proc. Natl. Acad. Sci. USA.

[183] Sykes P.J., Neoh S.H., Brisco M.J., Hughes E., Condon J., Morley A.A. (1992). Quantitation of targets for pcr by use of limiting dilution. Biotechniques.

[184] Pinheiro L.B., Coleman V.A., Hindson C.M., Herrmann J., Hindson B.J., Bhat S., Emslie K.R. (2012). Evaluation of a droplet digital polymerase chain reaction format for DNA copy number quantification. Anal. Chem..

[185] Hindson B.J., Ness K.D., Masquelier D.A., Belgrader P., Heredia N.J., Makarewicz A.J., Bright I.J., Lucero M.Y., Hiddessen A.L., Legler T.C. (2011). High-throughput droplet digital pcr system for absolute quantitation of DNA copy number. Anal. Chem..

[186] Dingle T.C., Sedlak R.H., Cook L., Jerome K.R. (2013). Tolerance of droplet-digital pcr vs real-time quantitative pcr to inhibitory substances. Clin. Chem..

[187] McNaught A.D., Wilkinson A. (1997). Compendium of Chemical Terminology: Iupac Recommendations.

[188] Massart D.L. (1997). Handbook of Chemometrics and Qualimetrics.

[189] Bustin S.A., Benes V., Garson J.A., Hellemans J., Huggett J., Kubista M., Mueller R., Nolan T., Pfaffl M.W., Shipley G.L. (2009). The miqe guidelines: Minimum information for publication of quantitative real-time pcr experiments. Clin. Chem..

[190] Kirschner M.B., van Zandwijk N., Reid G. (2013). Cell-free microRNAs: Potential biomarkers in need of standardized reporting. Front Genet..

